# Radiological and anatomical evaluation of the internal venous system in the context of access to the third ventricle - proposal of a new classification

**DOI:** 10.1007/s00701-025-06431-9

**Published:** 2025-01-24

**Authors:** Karol Zaczkowski, Piotr Łabętowicz, Małgorzata Podstawka, Rafał Wójcik, Ernest Jan Bobeff, Nicole Zielińska, Bartosz Szmyd, Manuel de Jesus Encarnacion Ramirez, Issael Ramirez, Łukasz Olewnik, Dariusz Jan Jaskólski, Karol Wiśniewski

**Affiliations:** 1 Department of Neurosurgery and Neurooncology, Medical University of Lodz, Barlicki University Hospital, Lodz, Poland; 2 Department of Forensic Medicine, Pathology and Histology, Masovian Academy in Plock, Plock, Poland; 3 Department of Clinical Anatomy, Masovian Academy in Plock, Plock, Poland; 4 Neuroscience Department, Instituto Nacional de Cancerologia, México city, México; 5 Department of Neurosurgery Oncology and Radiosurgery, CDD Private Hospital, Santo Domingo, Dominican Republic; 6 Department of Sleep Medicine and Metabolic Disorders, Medical University of Łódź, Łódź, Poland

**Keywords:** Internal cerebral vein, Deep cerebral veins, Susceptibility weighted imaging, Monroe foramen, Ventricular system

## Abstract

**Background:**

The internal venous system of the brain is a crucial anatomical landmark during accesses to the third ventricle through the foramen of Monro. Many classifications based on radiological assessment of the system have been developed, but they tend to be descriptive and do not highlight favorable anatomical variants. The aim of our study was to create a system based on morphometric measurements to facilitate preoperative decision-making regarding access to third ventricle tumors.

**Methods:**

We conducted an analysis of 119 MRI scans with SWI sequence using BrainLab software to create a model of the ventricular system, which allowed us to perform radiological measurements. We then validated these findings anatomically using 32 human brain specimens. The analyzed structures included the foramen of Monro (FM), the anterior septal vein (ASV), the thalamostriate vein (TSV), the venous angle (VA), the internal cerebral vein (ICV), and the distance between the FM and VA.

**Results:**

Based on the radiological analysis, we identified 9 internal venous systems, accounting for variations in each analyzed structure. The statistical analysis revealed no differences in the frequency of subtypes between radiological and anatomical studies (*p* = 0.097), nor in the occurrence of false venous angles (*p* = 0.520). We identified venous configurations that, in our assessment, are unfavorable in the context of accessing the third ventricle.

**Conclusion:**

The resulting classification accounts for significant clinical anatomical variations and, for the first time, provides specific morphometric values for each anatomical subtype. Consequently, it serves as a reproducible reference framework for preoperative planning of access to the third ventricle.

**Supplementary Information:**

The online version contains supplementary material available at 10.1007/s00701-025-06431-9.

## Introduction

The internal venous system (IVS) serves as a valuable set of surgical landmarks, providing critical information about the location of the foramen of Monro (FM) and the choroidal fissure. These veins play a crucial role in identifying the FM during a transforaminal approach to the third ventricle. This method is primarily used for removing lesions in the anterior and middle portions of the third ventricle, especially when the FM’s anatomy is altered due to a mass or hydrocephalus. The internal cerebral vein (ICV) is formed by the confluence of the thalamostriate vein (TSV) and the anterior septal vein (ASV). Their point of convergence was first defined as the venous angle (VA) in 1953 by Krayenbühl and Richter [14]. The VA is very important from the surgical point of view, especially during operations within the third ventricle, as it is often located in close proximity to the FM. According to the literature, in about 25–30% of cases, the VA forms at some distance from the posterior margin of FM, but no one has ever specified the exact distance between those anatomical structures. When the VA forms away from the FM, it is referred to as a “false venous angle” [12]. From the operator’s perspective, it is more advantageous to have this anatomical variant, as it allows for the dissection of the choroidal fissure posteriorly to the foramen of Monro, thereby improving the surgical view of the third ventricle [9].

Advances in brain radiological imaging provide a non-invasive imaging technique referred to as susceptibility-weighted imaging (SWI) that is well suited to assess veins. SWI is a 3D high-spatial-resolution fully velocity-corrected gradient-echo MRI sequence [6, 7]. SWI takes advantage of the effect of signal phase as well as its magnitude [6]. Compounds that have paramagnetic, diamagnetic, and ferromagnetic properties all interact with the local magnetic field distorting it and thus altering the phase of signal received from local tissue. Typically, the images presented in SWI depend on both signal magnitude and its filtered phase (combined post-processed magnitude and phase). Minimum intensity projection (minIP) presents conventional SWI images as a thick slab thus allowing to demonstrate better the venous anatomy [5]. SWI allows for identification of small amounts of haemorrhage/blood products or calcium, that cannot be visualized on other MRI sequences, as well as veins since deoxyhemoglobin present in venous blood changes the signal causing a loss of its magnitude and shifting its phase.

The ICV collects blood from the deep structures of the brain. Patients who experience damage to this vessel may present with drowsiness, hemiplegia, mutism, haemorrhagic infarction of the basal ganglia, or even death [11]. For this reason, a detailed knowledge of anatomical variations is essential. Although several classification proposals have been developed in the literature, some of them are based on CT-angiography studies, which are not routinely performed in patients with third ventricle lesions [3, 13]. Furthermore, these classifications are either based on anatomical or radiological studies, with none combining both methods. Additionally, the anatomical studies that form the basis of the classifications do not relate morphometric measurements to specific subtypes, making them more of a general guide and potentially prone to misinterpretation due to the lack of a reference point [4, 12, 15]. Therefore, we decided to create a classification that will primarily rely on radiological measurements, confirmed by morphometric measurements on human brain specimens. Additionally, we determined that our classification should focus primarily on the vessels that are most significant in the context of approaches to the third ventricle, ensuring its primary applicability in neurosurgical practice.

## Materials and methods

### MRI technique

We chose MRI images that were done in patients presenting with headaches. We used Siemens 3T MAGNETOM Trio scanner - T1-weighted sagittal MPRAGE (TR = 2400 ms, TI = 1000 ms, TE = 3.14 ms, FA = 8°, 0.9 mm^3^) voxels and a T2-weighted fast spin echo (TR = 3200 ms, TE = 469 ms, 1.0 mm^3^ voxels). Susceptibility weighted imaging sequence parameters for 3 T Siemens 3T MAGNETOM Trio scanner. Slabs 1, Dist. factor 20%, Position L0.0 A16.0 H37.8, Orientation T > C-6.9, Phase enc. dir. R > > L, Rotation 90.00 deg, Phase oversampling 0%, Slibe oversampling 23.10%, Slices per slab 104, FoV read 230 mm, FoV phase 75%, Slice thickness 1.50 mm, TR 28 ms, TE 20.00 ms, Averages 1, Concatenations 1, Filter Prescan normalize, Matrix size 256 × 256, TA 4.44, PAT 2, Voxel size 1.0 mm × 0.9 mm × 1.5 mm, Flip angle 15 deg, Dimension 3D, Bandwidth 120 Hz/Px, Slice resolution 100%, Coil elements HEA; HEP; NE1, 2.

## Visualization of FM in brainLab software

**Fig. 1:**
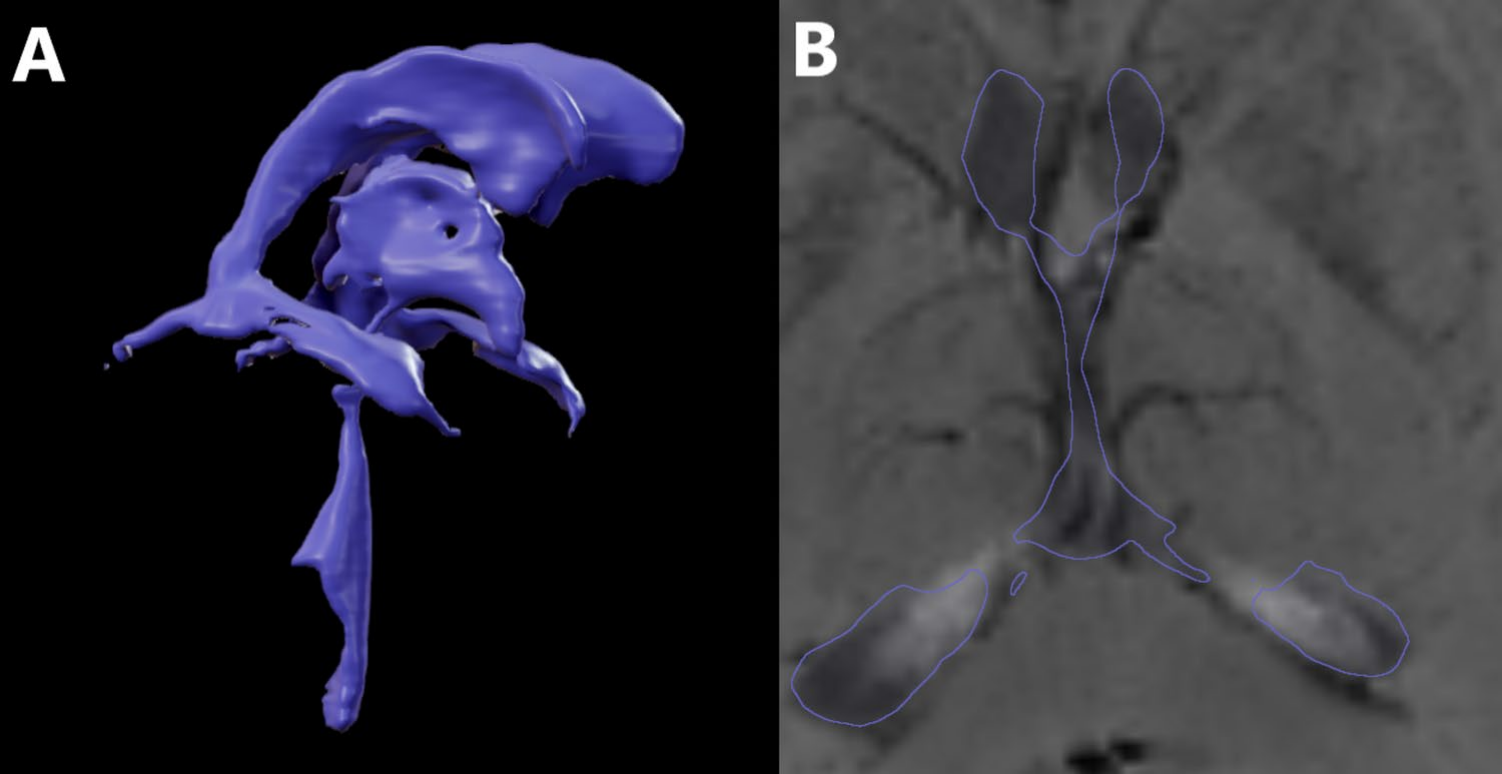
Part (**A**) shows a model of the ventricular system created using BrainLab software. Part (**B**) shows the model superimposed on a transverse plane of magnetic resonance SWI sequence

BrainLab software was used in this section for all steps. First, we merged T1, T2 and SWI MRI sequences and then performed automatic brain atlas segmentation. Secondly, from the anatomical atlas we chose only the 3D reconstruction of the ventricular system. That approach provided information about patient-specific 3D location of the FM. The anatomical atlas segmentation is a repeatable math algorithm, thus the data of every patient are processed in the same way Fig. 1.

### Radiological assessment of the deep vein pattern in the region of foramen Monroe and measurement of the distance between centre of foramen of Monroe and deep vein junction

We examined **119** brain MRI scans. As aforementioned, for each scan we performed 3D reconstruction of the ventricular system using BrainLab software. Then the 3D models (reconstructions based on BranLab automatic brain atlas segmentation) were merged with the SWI sequence. In the next step, we measured the distance between the center of the FM and the VA. Subsequently, we also investigated the variability of the deep veins in the region of the FM in order to select the most common patterns .

### Anatomical analysis of the deep vein pattern and the distance between centre of foramen of Monroe and deep vein junction

**Fig. 2:**
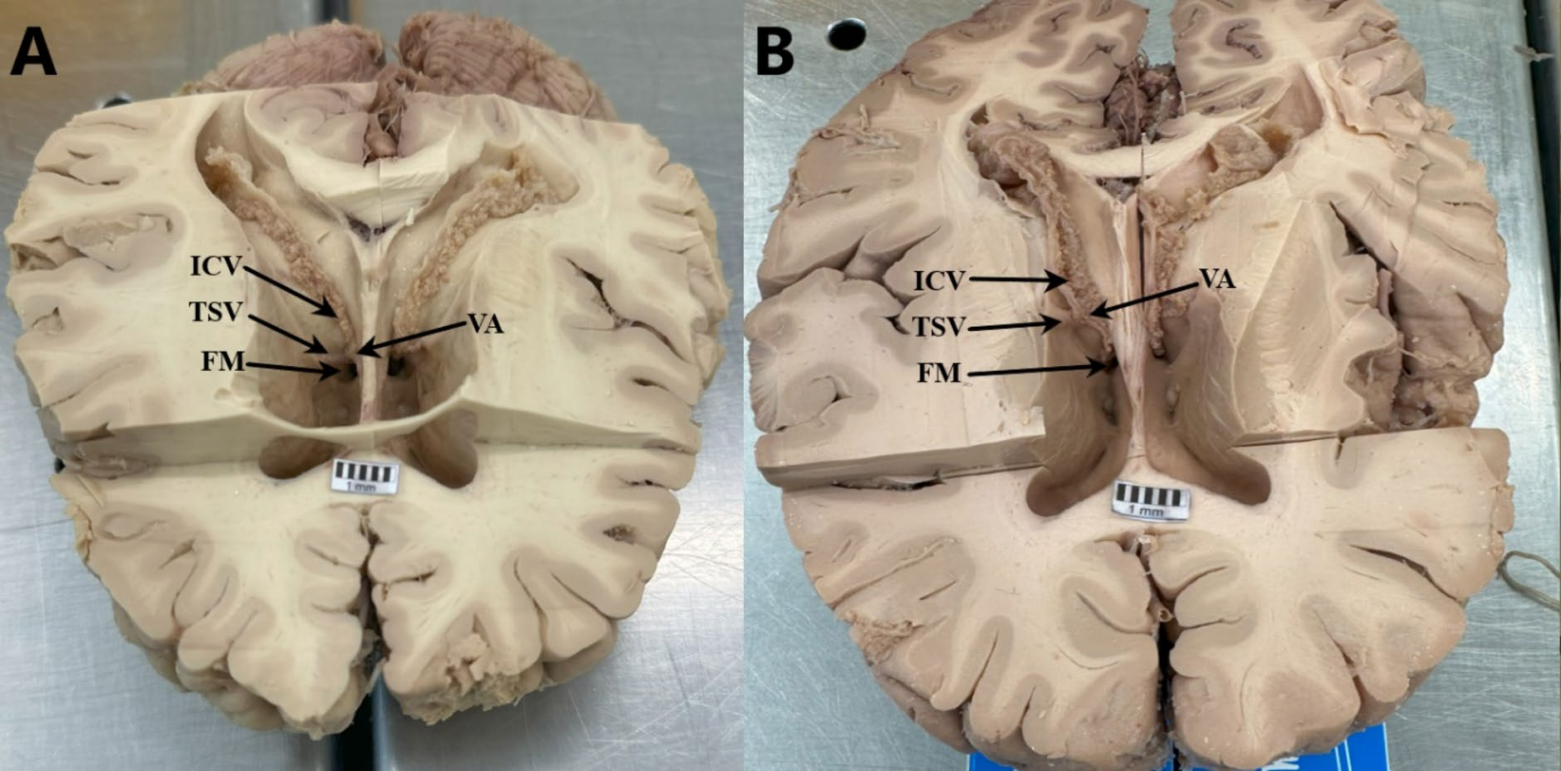
Shows example anatomical specimens. Part (**A**) indicates subtype IB, where the VA is formed within the lumen of the FM. Part (**B**) indicates subtype IA, where the VA is located nearby but does not contact the lumen of the FM. In both cases, the ICV is covered by the choroid plexus. ICV – Internal cerebral vein; TSV – Thalamostriate vein; VA – vein angle; FM – foramen of Monroe

In order to confirm radiological findings, we examined 32 (64 hemispheres) of human brain specimens. All specimens were fixed in a 10% solution of formalin for no less than 3 weeks. Our target was to evaluate internal cerebral veins and their distance to the FM. To expose the lateral ventricle, we conducted a transversal cut through the corpus callosum and parenchyma exposing the floor of the lateral ventricles. The internal cerebral vein runs in the ceiling of the third ventricle. To examine the distance between the internal cerebral vein junction and the centre point of the FM and to analyze the vessel patterns, we performed a micro-dissection of the choroid plexus. The measurements were done 3 times to get the arithmetic mean. In order to make our measurements more precise we took a photo of every specimen with a 10 mm scale and used a computer program to confirm the survey Fig. 2.

### Statistical analysis

Due to the numerous subgroups and the fact that some patterns occur significantly less frequently than others, statistical inference in this study is limited. To assess whether there are differences in the frequency of venous angle occurrence and specific patterns between the radiological and anatomical groups, Fisher’s exact test was performed. Due to the small sample size, the anatomical and radiological results are presented as median values with interquartile ranges (IQR) or simply as medians. Statistical analysis was conducted using IBM SPSS Statistics v27.0.

### Ethical approval

The study protocol was created according to the Declaration of Helsinki and Good Clinical Practice (GCP). It was further approved by the local Ethical Committee (number RNN/230/21/KE).

## Results

**Fig. 3:**
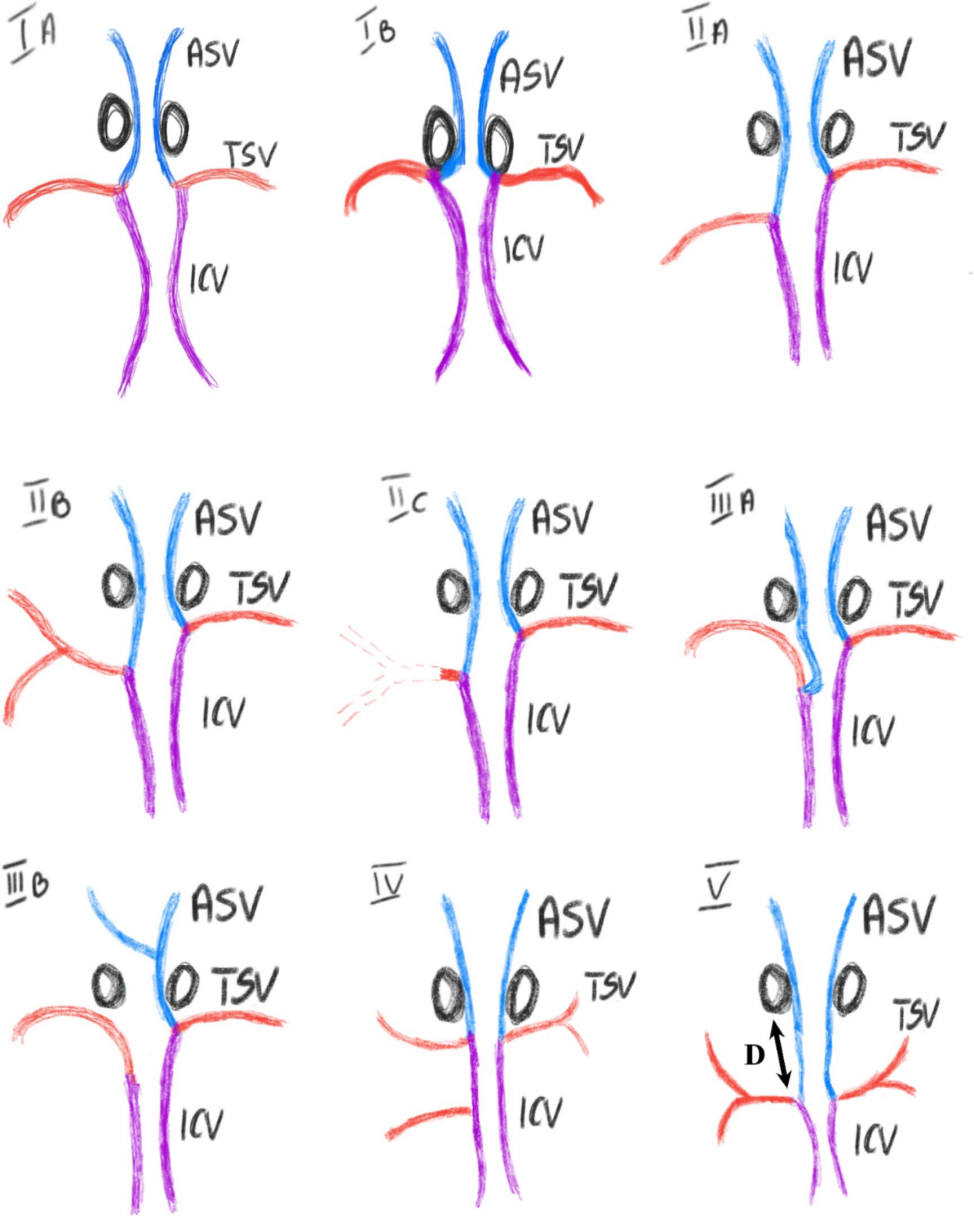
Presents the classification of the brain’s internal venous system, divided into groups and subgroups Group I: In this group, the VA forms bilaterally and symmetrically near the FM Subtype IA is characterized by the formation of the VA in close proximity to the FM but without direct contact with it Subtype IB is characterized by the formation of the VA within the lumen of the FM Group II: In this group, the IVS is variable depending on the TSV. In each subtype within this group, the VA on one side is formed at a significant distance from the FM Subtype IIA: The TSV is formed as a single vessel collecting blood from the entire striatum, and its course can be traced along the floor of the lateral ventricle Subtype IIB: The TSV is formed from an upper and lower branch that collects blood from the striatum. Both branches can be traces along the floor of the lateral ventricle Subtype IIC: The TSV is formed as a single vessel, but its course cannot be traced along the floor of the lateral ventricle, and therefore, it does not form a visible VA Group III: In this group, the variation depends on the variability of the ASV Subtype IIIA: In this pattern, neither the ASV nor the VA is visible along the floor of the lateral ventricle. The ASV runs within the brain parenchyma and connects with the TSV from below. The location of the VA formation relative to the FM is variable Subtype IIIB: In this pattern, the ASV is an unpaired vessel, collecting blood from the opposite side, forming a single venous angle Subtype IV: In this pattern, on at least one side, the TSV consists of upper and lower branches that do not form a single trunk. It does not belong to Group II because the upper trunk, together with the ASV forms the venous angle close to the foramen of Monro Subtype V: In this pattern, VA is formed by the ASV and TSV symmetrically and at a significant distance (D - >5 mm) from the FM; Radiological findings 119 SWI MR scans have been examined in order to assess the distance between FM and proximate vascular limitation. As the patterns of deep cerebral veins are highly variable, this resulted in limitations to the access to the third ventricle approach via the FM

Based on radiological analysis, followed by confirmation in studies on human brain specimens, we identified 9 distinct venous systems. The proposed classification incorporates elements from those available in the literature, but it expands on them by including previously omitted clinical subtypes that we consider significant. The subtypes, along with descriptions explaining their variability, are presented in Fig. 3.

### Radiological findings

**Fig. 4:**
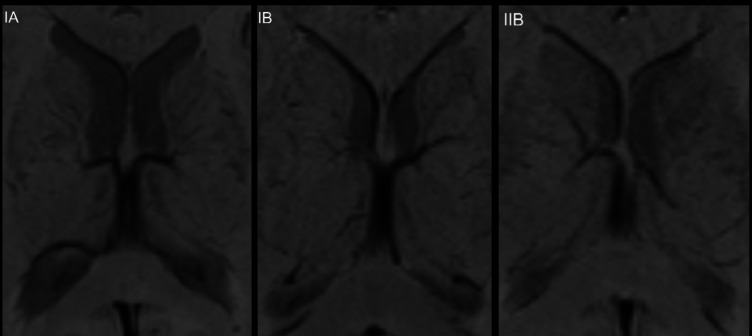
Presents example internal vein patterns in SWI sequence. All pattern are presented in Supplementary Material 1.

119 SWI MR scans have been examined in order to assess the distance between FM and proximate vascular limitation. As the patterns of deep cerebral veins are highly variable, this resulted in limitations to the access to the third ventricle approach via the FM. Figure 4 presents example internal vein patterns in SWI sequence. All pattern are presented in Supplementary Material 1 Table 1.

**Table 1:** Provides radiological summary of the prevalence of each venous subtype and their average distance from the FM to VA and the diameter of the FM

Subtype	Distance FM-VA-L (mm)	Distance FM-VA-*R* (mm)	FM diameter -L (mm)	FM diameter -*R* (mm)	Frequency of pattern occurrence (*n*%)
IA	2,94 (IQR:2,1–4,2)	2,79 (IQR: 1,89 − 4,3)	1,8 (IQR:1,40 − 2,35)	1,8 (IQR: 1,44 − 2,14)	31 (26,1%)
IB	1,67 (IQR: 1,16 − 3,06)	1,63 (IQR:1,20 − 2,80)	2,09 (IQR 1,55 − 3,02)	1,97 (IQR: 1,53 − 3,3)	21 (17,6%)
IIIA	5,46 (IQR:1,19 − 8,2)	3,81(IQR:1,88 − 7,69)	1,42 (IQR: 1,2–2,08)	1,38 (IQR: 1,03 − 2,41)	14 (11,8%)
IIIB	3,98	4,6	5,1	4,2	2 (1,7%) **
IV	2,64 (IQR:1,56 − 3,22)	2,94 (IQR:1,72 − 3,42)	1,36 (IQR:1,17 − 1,9)	1,46 (IQR:1,15 − 2,07)	10 (8,4%)
V	7,8	8,1	2,2	2,9	3 (2,5%) **
**Subtype**	**distance on the side with an FVA**	**Distance with normal VA**	**FM diameter -L** **(mm)**	**FM diameter -R (mm)**	**Frequency of pattern occurrence (n%)**
IIA*	2,91 (IQR: 2,2–4,4)	1,83 (IQR: 1,3–3,29)	1,73 (IQR: 1,23 − 2,1)	1,72 (IQR:1,245-2,1)	27 (22,7%)
IIB*	8,05 (IQR: 2,04–12,6)	2,96 (IQR: 2,34 − 4,6)	2,1 (IQR: 1,97 − 2,75)	2,1 (IQR: 1,59 − 2,74)	9 (7,6%)
IIC*	9,3	4,85	3,1	2,7	2 (1,7%) **

*n* = 119

*FVA* – False venous angle

*VA* – Vein angle

*FM* – Foramen of Monroe

* The average distance does not accurately reflect the typical distance for this subtype, as the venous angle, being distant from the foramen of Monro, occurs with similar frequency on both sides. Therefore, the table provides values for side with FVA and VA

** - median values

### Anatomical findings

We analyzed human brain specimens, and the data regarding the analyzed values are presented in Table 2. The presence of each radiological subtype has been confirmed at least once in sectional studies.

**Table 2:** Provides anatomical summary of the prevalence of each venous subtype and their average distance from the FM to VA and the diameter of the FM

Subtype	Distance to VA-L (mm)	Distance to VA-*R* (mm)	FM diameter -L (mm)	FM diameter -*R* (mm)	Frequency of pattern occurrence (*n*%)
IA	3,45 (IQR:3,23 − 3,62)	3,31 (IQR: 3,2–3,7)	2,61 (IQR:2,48 − 2,79)	2,63 (IQR: 2,44 − 2,84)	12 (37,5%)
IB	2,44 (IQR: 1,89 − 2,66)	2,42 (IQR:2,00–2,58)	2,7(IQR: 2,15 − 3,56)	3,26 (IQR:2,43 − 3,45)	8 (25%)
IIIA	3,56	3,12	3,95	3,5	2 (6,3%) **
IIIB	3,9	4,6	1,7	2,3	1 (3,1%)
IV	5,0	7,0	1,7	3,3	1 (3,4%)
V	6,5	8,75	1,67	3,3	3 (9,4%) **
**Subtype**	**Distance on the side with an FVA**	**Distance with normal VA**	**FM diameter -L** **(mm)**	**FM diameter -R (mm)**	**Frequency of pattern occurrence (n%)**
IIA*	3,45	6,75	2,93	3,14	2 (6,3%) **
IIB*	5,07	3,93	3,05	2,83	2 (6,3%) **
IIC*	5,9	4,0	3,1	2,7	1 (3,1%)

*n* = 32

*FVA* – False venous angle

*VA* – Vein angle

*FM* – Foramen of Monroe

* The average distance does not accurately reflect the typical distance for this subtype, as the venous angle, being distant from the foramen of Monro, occurs with similar frequency on both sides. Therefore, the table provides values for side with FVA and VA

** - median values

### Statistical analysis

No differences were observed in the occurrence of the "false venous angle" between the anatomical and radiological groups (p=0.520). Additionally, the frequency of each pattern did not differ between the anatomical and radiological groups (p=0.097).

## Discussion

The primary aim of our study was to identify subtypes of the IVS and relate them to the classifications known from the literature. We identified 9 types of venous systems, which were divided into subgroups to clearly and distinctly highlight the differences between each subtype. Additionally, our study confirmed the usefulness of the SWI sequence, as documented in the literature, for evaluating the IVS of the brain [13]. The decision to create a new classification came from our belief that currently there is no system that fully mirrors the anatomical variability of this region. In 2019 Brzegowy et al. [3] proposed a classification that defines the point of VA formation unilaterally, however we believe that its use can be problematic for operators who must consider the brain’s symmetry and as demonstrated in this study, there are instances of IVS where one side remains dependent on the other. Furthermore, although it accounts for the VA position relative to the FM, it does not specify the exact distances. It is also important to note that the 2019 classification is based on angio-CT imaging, which is not routinely performed in patients with third ventricle tumours. Therefore, in our view, a classification based on MRI with the SWI sequence, with subtypes confirmed in anatomical studies, and particular morphometrical parameters, offers a better approach.

The creation of this classification was not an end in itself but rather an attempt to answer the question of which anatomical variants might potentially influence approaches to the third ventricle and, if so, how. Group I includes the most common anatomical variants, among which subtype IB is considered unfavorable for performing a transcallosal transforaminal approach. This is because, if the lesion does not sufficiently enlarge the FM from the operator’s perspective, its expansion could be risky due to the VA position within its lumen. For this reason, the transchoroidal approach seems to be safer. Subtype IA is slightly more favorable than IB, as it allows for a small expansion of the FM if necessary. For the entire first subgroup, the authors believe that alternative approaches, such as transcallosal transchoroidal, should be considered, which is consistent with the literature describing these approaches [12, 16]. Group II represents the venous configurations for which the transcallosal transforaminal approach may be the most advantageous. Since the VA forms at a considerable distance from the FM, this setup allows for relatively unrestricted expansion of the foramen. However, this configuration is slightly less favorable for transchoroidal approaches because the VA is formed within the choroidal fissure. In subtype IIA, expanding the FM is the safest since the upper branch of the TSV is not present. The enlargement of the FM in subtype IIB is slightly less favorable due to the upper branch, which often limits the expansion. Subtype IIC requires particular caution because, upon entering the lateral ventricle, it is not possible to trace the TSV. This makes expanding the FM, though possible, significantly less safe compared to subtypes IIA or IIB. The essence of Group III is the variability of the ASV. In subtype IIIA, the ASV forms the VA posterior to the FM; however, it is not possible to precisely identify the point of the angle’s formation, as the ASV angles with the TSV from below, penetrating the tissue. In such cases, performing a transchoroidal approach could lead to damage of ASV, making the transforaminal approach preferable for this group. In subtype IIIB, the ASV forms a VA on only one side, which means that the transchoroidal approach might be safer on the side without the angle. Subtype IV is a rare subtype but is considered the least favorable for approaches through the lateral ventricle. This is because both the transforaminal approach is limited due to the close formation of the VA, and the transchoroidal approach is constrained by the varying connection points of the upper and lower branches of the ASV with the ICV. Subtype V is advantageous for transforaminal approaches because the VA are symmetrically formed at a considerable distance from the FM. Analyzing the IVS is crucial when planning approaches involving the lateral ventricle. However, it is essential to remember that there are other potential entry points to the anterior part of the lateral ventricle, such as through the lamina terminalis or between the columns of the fornix [10, 16]. Each method carries certain risks, and our classification aims to help the operator assess these risks and facilitate decision-making. Furthermore, defining specific morphometric parameters, such as the distance between the venous angles, ensures that this classification can be implemented and that the conclusions from the anatomical study can be applied using commonly available tools in any neurosurgery department.

According to the available literature, our study is the first in which radiological subtypes have been anatomically validated. We consider this as a significant strength of our research which allows for a more accurate determination of the relationship between the venous system and the FM. We are also the first to define the distances of the venous angle and its formation relative to the FM within a classification system. This provides surgeons with critical information, enabling to anticipate the anatomical conditions they will face at surgery.

It should be noted that our system, to facilitate interpretation for the surgeon, focuses on the ASV TSV, VA (Venous Angle), and ICV as the most critical components of the brain’s internal venous system. These vessels are fundamentally the most significant in a clinical context. Including variants that account for the medial atrial veins (MAV) and lateral direct veins (LDV) would have significantly expanded the system with only minimal clinical impact from the described variations. Additionally, the approach of describing the venous system by focusing solely on these vessels has been previously used in the literature [5]. While developing this classification, our aim was to identify the most common variations within the vessels considered. The presence of nine subtypes is due to the high degree of anatomical variability observed. Only with this number of subtypes were we able to delineate the most frequent variants, ensuring that intra-group and intra-subtype differences were minimal and did not overlap with the findings of other groups.

Compared to classifications based on anatomical and radiological studies [4, 12], our system is more detailed. The previous systems mainly focused on the location of the FM relative to the VA, whereas we considered each vessel forming the VA and accounted for their variability. We believe this is a more complete approach, as limiting the focus to the point of VA origin may lead to damage to the TSV branches and, in some cases, to the ASV running through the parenchyma. The morphometric values in previous studies are similar to ours but difficult to relate, as the distances were provided in a general manner without reference to specific subtypes.

A similar classification system to ours was developed in 2010 by Fujii et al. [5]. In a study involving MRI scans of 321 patients, they analyzed variations within the ASV, TSV, and ICV veins. However, this system is based on only four subtypes, with all other variations grouped under subtype III, which, in our opinion, inadequately captures the complex anatomy of this region. Moreover, similar to the 2019 classification, it does not specify the actual distance of the VA from the FM. This limitation makes the system more subjective, with the classification into a specific subtype relying more on the evaluator’s judgment than on an objective measurement of distance. Although distances between specific anatomical landmarks were radiologically determined in other studies [15], they have not been evaluated anatomically.

Using widely available tools we may create a good preoperative visualization of patient-specific distance between FM and the junction of deep internal veins. This helps us to choose an appropriate approach in each patient. It is important to note, however, that tumors of the third ventricle can alter the anatomy of the internal veins. Therefore, this system and its findings should be considered as an auxiliary tool during the preoperative stage. For example, the transcallosal-transforaminal approach is preferred when the foramen of Monroe is enlarged by the tumour or hydrocephalus. The anterior part of corpus callosum (CC) can be dissected without any neurological consequences, if the incision is made up to 22 mm in long axis of CC [1]. Although, in case of third ventricle tumour without dilatation of the FM we may chose transcallosal-transfornical approach, this caries a risk of fornix column damage and impair memory. Using these tools, we may check the location of the deep vein junction and in case of favorable anatomical conditions, choose transcortical-transforaminal approach. If the FM is enlarged posteriorly along the choroidal fissure, transchoroidal [2] or subchoroidal [8] approach may be more preferred. We believe that our radiological studies, supported by anatomical data, along with the widespread accessibility of the methodology used in this work, make this classification potentially significant for preoperative planning.

A significant limitation of our study is the relatively small number of specimens examined (32). Although this number is limited, particularly when studying subtypes that can be extremely rare, we were able to confirm the anatomical presence of each of these subtypes. However, it should be noted that some subtypes were found only once (IIIB, IV, and IIC), which prevents meaningful comparison of morphometric radiological and anatomical data. The validation was particularly challenging for subtypes IIC and III, due to the intraparenchymal course of the vessels. Another limitation is that brain specimens require appropriate injection, which affects their consistency and may slightly distort distances. However, we did not observe any significant impact of the injections on differences in the patterns. Although MRI devices are becoming widely available worldwide, not all centers have the capability to perform high-quality MR scans with SWI sequences. In such situations, the interpretation of the classification may be challenging, which is a significant limitation of this system. An additional issue may be the inaccuracy of the ventricular system segmentation processes in BrainLab software. However, in our opinion, this will not affect the interpretation of specific subtypes but may result in a slightly higher margin of error in the already approximate morphometric assessment.

It is important to mention that, based on the data available in the literature, our study still appears to be the largest anatomical study of the internal venous system to date.

## Conclusion

We propose a new classification for the IVS based on radiological assessment, anatomical validation, and specific morphometric values. We believe it could become a valuable tool in the preoperative planning for patients with third ventricular tumours, thereby enhancing patient safety.

## Supplementary Information

Below is the link to the electronic supplementary material.

ESM 1 (PNG 624 KB )

High Resolution Image (TIF 1.19 MB)

## Data Availability

No datasets were generated or analysed during the current study.
