# Supplementary figures and images for: Radiological and anatomical evaluation of the internal venous system in the context of access to the third ventricle - proposal of a new classification
Source: Acta Neurochir (Wien). 2025 Jan 24;167(1):23. doi: 10.1007/s00701-025-06431-9 (PMC11761785; doi:10.1007/s00701-025-06431-9)

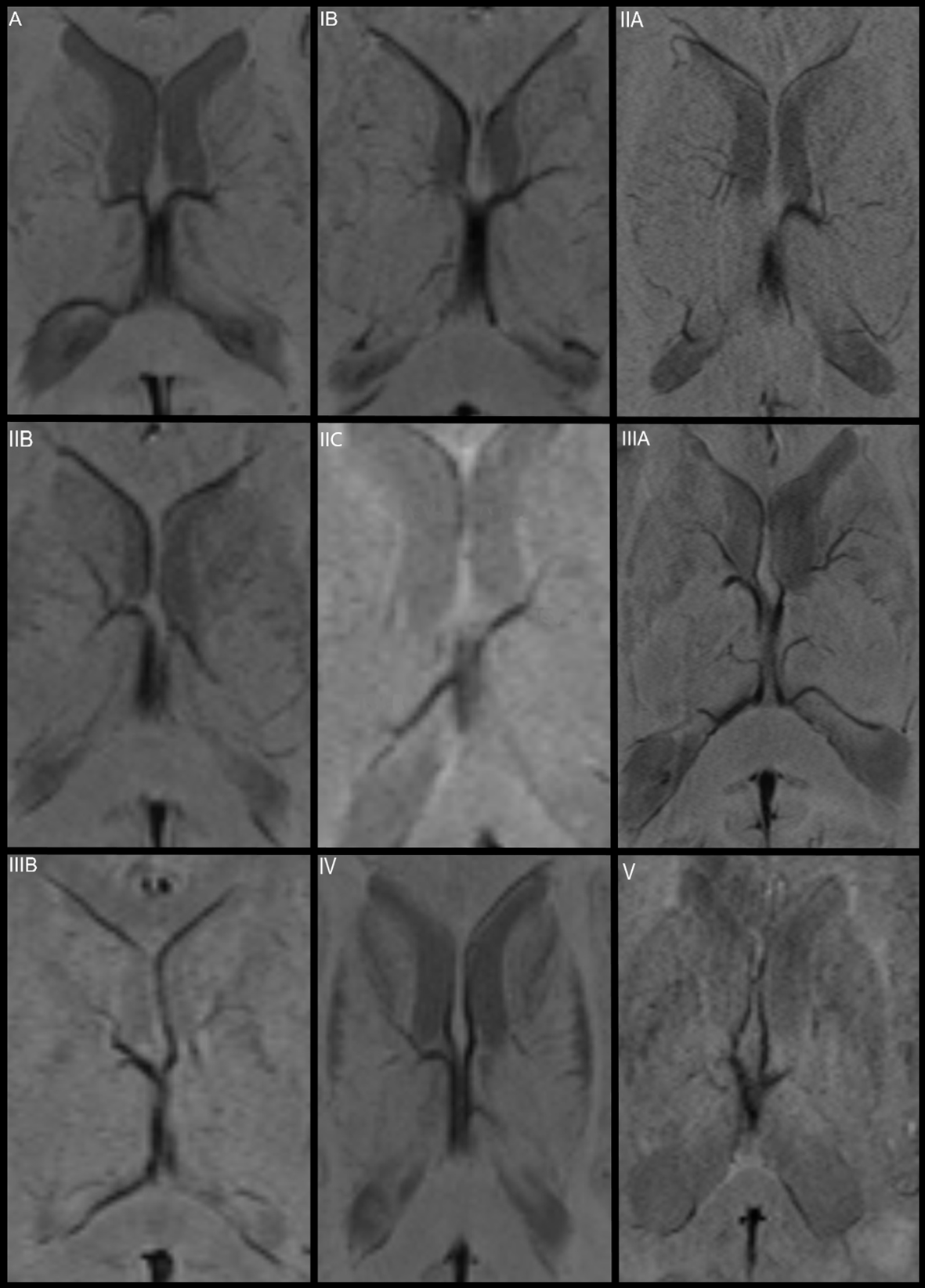

Supplement: Supplementary file 1 — (PNG 624 KB ) [file 701_2025_6431_Fig5_ESM.png]

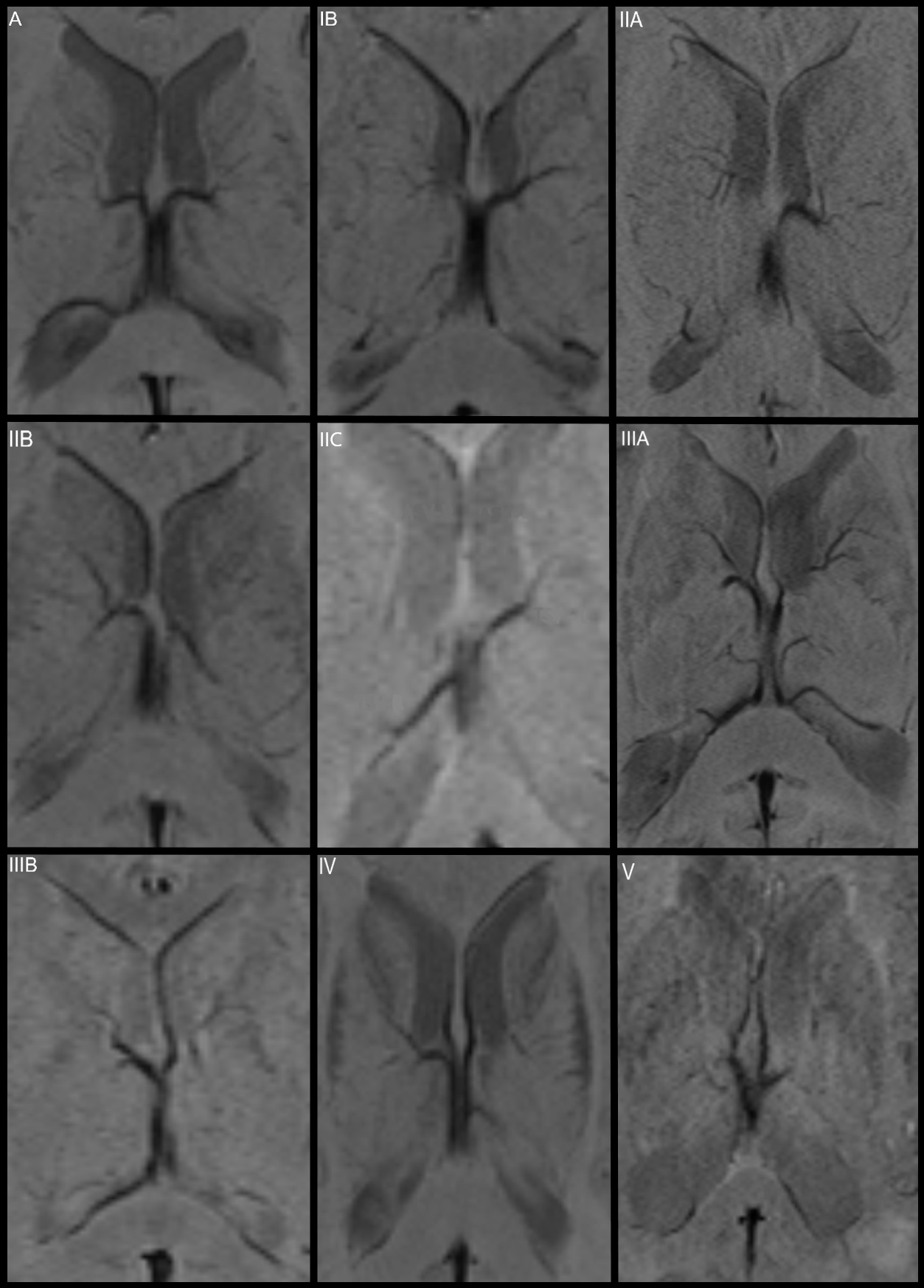

Supplement: Supplementary file 2 — High Resolution Image (TIF 1.19 MB) [file 701_2025_6431_MOESM1_ESM.tiff]
